# Efficacy of granulocyte and monocyte adsorptive apheresis on skin and joint manifestations of palmoplantar pustulosis with pustulotic arthro‐osteitis: A multicentric, prospective, observational study

**DOI:** 10.1111/1346-8138.17667

**Published:** 2025-02-12

**Authors:** Namiko Abe, Yuko Higashi, Chiharu Tateishi, Takuro Kanekura, Daisuke Tsuruta, Tomoko Kobayashi, Yukari Okubo

**Affiliations:** ^1^ Department of Dermatology Tokyo Medical University Tokyo Japan; ^2^ Department of Dermatology Kagoshima University Graduate School of Medical and Dental Sciences Kagoshima Japan; ^3^ Department of Dermatology Graduate School of Medicine Osaka Metropolitan University Osaka Japan

**Keywords:** clinical efficacy, granulocyte and monocyte adsorptive apheresis (GMA), palmoplantar pustulosis, pustulotic arthro‐osteitis

## Abstract

Granulocyte and monocyte adsorptive apheresis (GMA) selectively removes activated granulocytes and monocytes from the peripheral blood. In 2012, GMA was approved in Japan as a treatment for generalized pustular psoriasis and localized pustular psoriasis or palmoplantar pustulosis (PPP). Limited evidence from case reports and monocentric studies suggested that GMA is an effective treatment for skin and joint symptoms of PPP with pustulotic arthro‐osteitis (PAO). The present, prospective, observational study was performed at three dermatology departments in Japan to evaluate the efficacy and safety of GMA in patients with PPP with PAO. Between April 2017 and December 2020, two male and 12 female patients with PPP and PAO were enrolled. Their mean age, mean duration of skin manifestations, and mean duration of PAO symptoms was 52.8 years, 51.2 months, and 44.9 months, respectively. GMA was applied weekly over five sessions. The skin and joint symptoms were assessed at baseline, post‐GMA, and at the 3‐month follow‐up. A total of 12 patients completed five GMA sessions, and two patients discontinued the treatment because of adverse events. Thus, 12 patients were finally assessed post‐GMA, and 10 patients were assessed at the 3‐month follow‐up. The assessment of GMA efficacy demonstrated that the skin symptoms had remarkably improved and improved in 33.3% (4/12) and 70% (7/10) of the patients post‐GMA and at the 3‐month follow‐up, respectively. Furthermore, the joint symptoms had remarkably improved in 66.7% (8/12) and 60% (6/10) of the patients post‐GMA and at the 3‐month follow‐up, respectively. These results suggest that GMA is effective in treating the skin and joint symptoms of PPP with PAO.

## 1 INTRODUCTION

Palmoplantar pustulosis (PPP), also known as pustulosis palmaris et plantaris, is a chronic, inflammatory skin disorder presenting with sterile pustules, small blisters, erythema, and scales on the palms and soles. A nationwide survey^1^ found that the PPP prevalence in Japan was 0.12%, indicating that it is relatively common. Inflammatory arthro‐osteopathy is a significant complication of PPP and manifests in approximately 10%–30% of patients, with sternoclavicular arthritis, generally referred to as pustulotic arthro‐osteitis (PAO), being particularly common.^2^, ^3^

Recently, smoking and focal infections have been found to trigger or exacerbate PPP and PAO,^4^, ^5^, ^6^, ^7^ and smoking cessation, along with the treatment of focal infections, were found to affect treatment outcomes of both PPP and PAO favorably.^8^, ^9^, ^10^

In Japan, the current standard treatments of PPP and PAO include topical corticosteroids, vitamin D3, phototherapy, systemic therapy with non‐steroidal anti‐inflammatory drugs (NSAIDs), cyclosporine, etretinate, methotrexate, and biologics. However, these treatments have several limitations, owing to their insufficient efficacy, side effects, and intolerability.

Granulocyte and monocyte adsorptive apheresis (GMA) using Adacolumn® (JIMRO Co.) is an extracorporeal leukocyte apheresis device filled with cellulose acetate beads which selectively adsorb approximately 65%, 55%, and 2% of granulocytes, monocytes/macrophages, and lymphocytes respectively, from the peripheral blood.^11^, ^12^, ^13^

Granulocyte and monocyte adsorptive apheresis has been used clinically as a non‐pharmacological treatment and has demonstrated efficacy as a treatment for ulcerative colitis (UC), Crohn's disease (CD), psoriatic arthritis (PsA), and pustular psoriasis.^14^, ^15^, ^16^, ^17^ In their multicentric, single‐arm, prospective study, Ikeda et al. reported an 85.7% response rate to GMA in patients with GPP.^17^ In 2012, 1 year before the publication of their study, Adacolumn® was approved in Japan for use in patients with GPP and localized pustular psoriasis who were either unresponsive to, or were ineligible for, existing oral, systemic therapies. In Japan, PPP is considered to be a discrete, pathological entity while in the US and western Europe, it is classified as a localized form of pustular psoriasis; consequently, under Japan's National Health reimbursement scheme as well, PPP is treated as a localized pustular psoriasis. Nevertheless, since the aforementioned clinical trials did not enroll patients with localized pustular psoriasis,^17^ the efficacy of GMA in patients with PPP remained inconclusive. However, after GMA was approved for use in Japan, several studies reported its efficacy against the skin manifestations of PPP^18^, ^19^, ^20^, ^21^, ^22^, ^23^ as well as the joint symptoms of PPP with PAO,^19^, ^20^, ^21^, ^22^, ^23^ although all these studies were case reports or monocentric studies (Table 1). Hence, the present study aimed to evaluate the efficacy and safety of GMA for the treatment of the skin and joint symptoms of PPP with PAO in a multicentric setting.

**TABLE 1:** Patient characteristics, disease characteristics and therapeutic interventions before GMA.

Valuable	*n* = 14
Age, years, mean ± SD (min–max)	52.8 ± 14.1 (29–76)
Gender, male/female	M, 2; F, 12
PPP duration, month [*n* = 13], mean ± SD (min–max)	51.2 ± 53.5 (3–180)
PAO duration, month, mean ± SD (min–max)	44.9 ± 54.4 (0.5–180)
Smoking status	No smoking history, 3; smoking cessation, 7; smoking continuation, 2
Concomitant focal infection	Yes: 7, No: 7
Types of focal infection^a^	Periodontitis, 2; tonsillitis, 4; sinusitis, 2
Concomitant disease	Glaucoma and hay fever, 2; hypertension, hyperlipidemia, gastric ulcer, Alzheimer's disease, Hashimoto's disease, alopecia areata, insomnia, and Meniere's disease, 1
PPPASI, mean ± SD	9.7 ± 10.2
Site of arthritis, number (%) of patients	
Peripheral	0
Axial	12 (85.7)
Peripheral and axial	2 (14.3)
Osteoclastic or deformation, number (%) of patients	6 (42.9)
VAS for joint pain, mean ± SD	75.1 ± 24.4
Tender joint count (0–68), mean ± SD	7.6 ± 8.8
Swollen joint count (0–66), mean ± SD	4.9 ± 8.0
CRP, mg/dL, mean ± SD	1.2 ± 2.1
VASfor the physicians' global assessment of joint, mean ± SD	74.9 ± 14.1
VASfor the patient's global assessment, mean ± SD	63.8 ± 26.8
HAQ, mean ± SD (*n* = 12)	6.2 ± 7.2
Therapeutic interventions	
Systemic treatment	NSAIDs, 11; methotrexate, 2; cyclosporine, 1; other pain mitigation medications, 4; antihistamines, 2; biotin supplements, 1; antibiotics, 1; herbal medicine, 1
Topical treatment	NSAIDs, 3; calcitriol/betamethasone propionate, 3; steroid, 2; vitamin D3, 2
Other treatment	Dental treatment, 6; tonsillectomy, 4

Abbreviations: CRP, C‐reactive protein; GMA, granulocyte and monocyte adsorptive apheresis; HAQ, HealthAssessment Questionnaire; NSAIDs, non‐steroidal anti‐inflammatory drugs; PAO, pustulotic arthro‐osteitis; PPP,  Palmoplantar pustulosis; PPPASI, palmoplantar pustulosis area and severity index; SD, standard deviation; VAS, visual analogue scale.

a One patinet had both tonsillitis and sinusitis.

## 2 METHODS

The present study complies with the Declaration of Helsinki and was approved by the institutional review board of Tokyo Medical University (approval nos. 2017‐046, 2018‐2019). Written informed consent was obtained from all the study participants before enrollment.

### 2.1 Patient population

The diagnostic specifications for PPP with PAO in the present study were based on the Sonozaki criteria:^2^ (i) the presence or past history of typical PPP skin manifestations; (ii) joint pain occurring particularly in the sternoclavicular joint or elsewhere coinciding with hyperostotic imaging findings indicative of PAO; (iii) joint symptoms persisting for at least 6 months after diagnosis; and (iv) the absence of other, orthopedic oriented arthropathy. Imaging studies included X‐ray, magnetic resonance imaging, computed tomography, and bone scintigraphy and imaging evaluation methods was performed based on the modified Sonozaki criteria.^24^ The inclusion criteria were (i) the presence of PPP associated with PAO manifesting skin and joint symptoms; (ii) age ≥16 years; (iii) history of the clinical use of GMA; (iv) intolerance or resistance to conventional, systemic treatments, including NSAIDs and disease‐modifying antirheumatic drugs (DMARDs). The exclusion criteria were (i) neutrophil count <2000/mm^3^, (ii) the presence of infectious complications; and (iii) ineligibility for study enrollment as deemed by the attending physician.

### 2.2 Treatment

Granulocyte and monocyte adsorptive apheresis was administered using Adacolumn® once a week over 5 weeks. One GMA session lasted 60 min, and the blood flow rate was 30 mL/min. Blood was accessed via one arm and returned to the patient from the column outflow through a venipuncture in the antecubital vein of the contralateral arm. Before each session, the apheresis system was primed by running 1 L of physiological saline followed by another liter of saline containing an anticoagulant. During the session, heparin, including low‐molecular‐weight heparin, or nafamostat mesylate was used as an anticoagulant in a continuous infusion. The typical heparin dosage was 4000 and 2000 U for system priming and continuous infusion, respectively. In contrast, the dosage of nafamostat mesylate was 20 and 30 mg for the respective procedures. The type of anticoagulant used was left to the discretion of the attending physician.

### 2.3 Study design

The present, multicentric, prospective, observational study was conducted at three dermatology departments in Japan from April 25, 2017 to December 31, 2020 to evaluate the efficacy and safety of GMA in patients with PPP and PAO, which were together considered to be a form of localized pustular psoriasis.

Patient data at baseline within 3 weeks of the first GMA session included age, sex, PPP duration, PAO duration, the Palmoplantar Pustulosis Area and Severity Index (PPPASI) score,^25^ 68 tender joint counts, 66 swollen joint counts, the patient's assessment of joint pain (mm) and their global assessment of arthritis (mm) on the visual analog scale (VAS), the Physician's Global Assessment (PGA)of arthritis (mm) on the VAS, Health Assessment Questionnaire (HAQ) results,^26^, ^27^ smoking status (current smoking, cessation, non‐smoking history), concurrent focal infections (tonsillitis, sinusitis, caries, periapical pathosis, etc.), history of tonsillectomy, current medications, current medical disorders, and imaging modality used to evaluate osteoarthritis. The evaluation of joint symptoms during the study followed the same protocol used by rheumatologists in the American College of Rheumatology core set to evaluate the 68 tender joints and 66 swollen joints including the sternoclavicular and sternocostal joints. The tender joints for each patient were evaluated by a physician. Tender joints were determined by whether the patient felt pain when the physician applied pressure to each joint, and swollen joints were determined by whether there was swelling when the physician visualized and palpated each joint.

### 2.4 Efficacy assessments

To assess the efficacy of GMA, patients were assessed at baseline within the 3 weeks before the first GMA session, within 2 weeks after the fifth GMA session, and at the 3‐month follow‐up after the first GMA session. The efficacy assessment criteria for the skin symptoms were quantified using the PPPASI score, while joint symptoms, were quantified using the following categories for joints: (1) VAS of the patient's assessment of arthritis‐related pain (mm), (2) tender joint count, (3) swollen joint count, (4) C‐reactive protein (CRP) test (mg/dL), (5) VAS of the patient's global assessment of arthritis (mm), (6) VAS of the PGA of arthritis (mm), and (7) the HAQ results.

Improvement in the skin symptoms at each time point was categorized as “remarkably improved” (≥75% from the baseline PPPASI score), “improved” (50%–75%), “unchanged” (<50%) or “deteriorated” (any deterioration from the baseline). Similarly, improvement in the joint symptoms was categorized as “remarkably improved” (improvement in all four items from 1 to 4), “improved” (improvement in two to three items), “unchanged” (improvement in one or no items or deterioration in one item) or “deteriorated” (deterioration in more than two items). Furthermore, to explain the effect of smoking status and the presence of concurrent focal infections on the efficacy of GMA for skin and joint symptoms, patients who were assessed as “Remarkably improved” or “Improved” were defined as the improved group and “improvement rate”, and “unchanged” or “deteriorated” was defined as the not improved group. The effect of smoking status and presence of concomitant focal infections on the rate of improvement was then evaluated.

### 2.5 Safety assessment

Patients who underwent at least one GMA session were assessed for any symptoms or unexpected events at each visit throughout the treatment period.

### 2.6 Statistical analysis

Continuous variables were expressed as the mean ± standard deviation. Categorical variables were expressed as a number and percentage. The Wilcoxon signed‐rank test was used to determine the difference between items at the baseline and each assessment (post‐GMA and at the 3‐month follow‐up). Additionally, the Fisher's exact test was used to assess the improvement rate by patient characteristics (smoking status, and concurrent focal infection).

The statistical software JMP Pro, version 14.0.0 (SAS Inc.) was used.

## 3 RESULTS

### 3.1 Patient disposition

In total, 14 patients with PPP and PAO from three institutions were enrolled. Table 1 shows a summary of the patients’ characteristics. Two patients were male and 12 were female, and their mean age was 52.8 ± 14.1 years. The mean duration of PPP and PAO was 51.2 ± 53.5 and 44.9 ± 54.4 months, respectively. In six patients, the onset of PPP preceded that of PAO; in three patients, the onset of PAO preceded that of PPP; four patients had simultaneous onset; and one patient had an unknown time of PPP onset. Table 1 shows a list of assessment items for the skin and joint symptoms at baseline and the therapeutic interventions undertaken pre‐GMA. Notably, none of the patients received biologics or Janus kinase (JAK) inhibitors The diagnostic imaging methods used to assess osteoarthritis included bone scintigraphy in 11 patients, magnetic resonance imaging in seven patients, X‐ray in seven patients, and computerized tomography in two patients. Some patients were evaluated using multiple imaging modalities. No patient had imaging studies performed after GMA.

### 3.2 Efficacy

**FIGURE 1:**
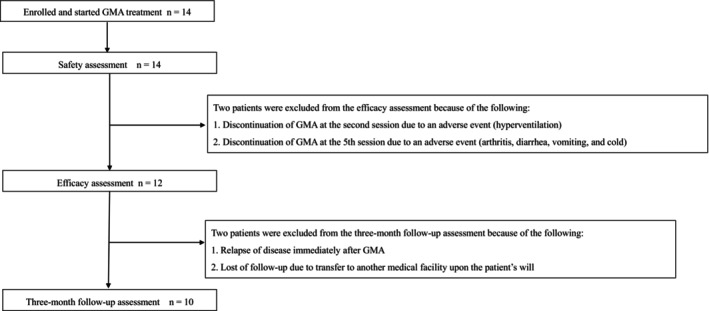
Patient flowchart.

Among the 14 patients who underwent GMA, 12 successfully completed the planned course of five GMA sessions while two had to discontinue because of adverse effects. Consequently, 12 patients were available for efficacy assessment post‐GMA. Furthermore, 10 patients received the 3‐month follow‐up assessment except one patient who experienced a relapse immediately after the GMA and one patient who elected to be transferred to another medical facility (Figure 1).

**FIGURE 2:**
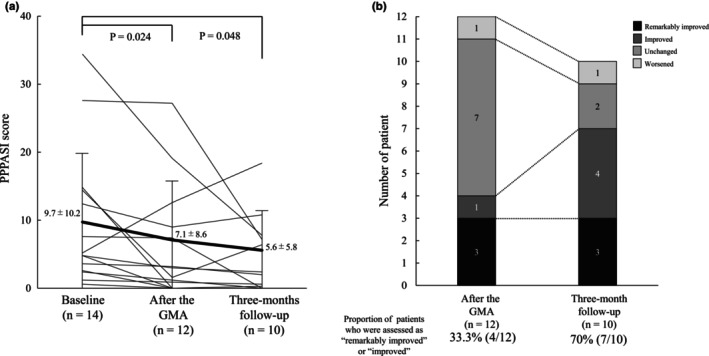
Assessment of efficacy of granulocyte and monocyte adsorptive apheresis (GMA) for skin manifestations. (a) Shifts in the palmoplantar pustulosis area and severity index (PPPASI) score at each assessment time point. The solid lines represent individual patients, and the bold line represents the mean PPPASI scores for all patients at each assessment time point. (b) Results of the efficacy assessment of GMA for skin manifestations using a four‐grade scale after the GMA and at the 3‐month follow‐up.

Figure 2a shows the PPPASI scores at baseline, post‐GMA, and at the 3‐month follow‐up. The PPPASI score significantly decreased from 9.7 ± 10.2 at baseline to 7.1 ± 8.6 post‐GMA (*p* = 0.024) and further significantly decreased to 5.6 ± 5.8 at the 3‐month follow‐up (*p* = 0.048). Among the 12 patients assessed for efficacy, three (25%) had remarkably improved skin symptoms post‐GMA and one (8.3%) had improved skin symptoms. In total, four (33.3%) patients received the assessment of remarkably improved or improved (Figure 2b).

**FIGURE 3:**
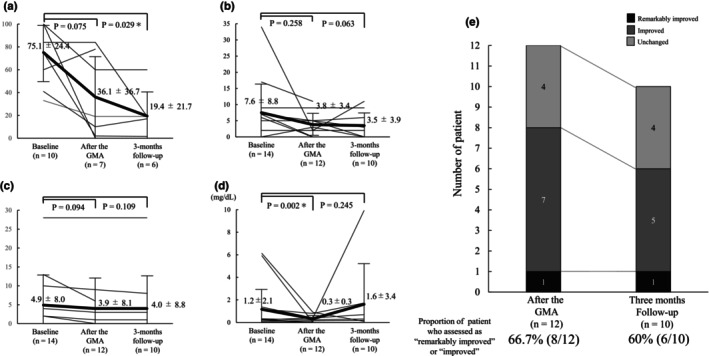
Efficacy assessment of granulocyte and monocyte adsorptive apheresis (GMA) for joint symptoms and C‐reactive protein (CRP). (a) Shifts in the visual analog scale of joint pain (mm) at each assessment time point. (b) Tender joint. (c) Swollen joints. (d) CRP (mg/dL). Solid lines represent individual patients, and bold lines represent the mean values for all patients. (e) Results of the GMA efficacy assessment for joints symptoms using a four‐grade scale after GMA and at the 3‐months follow‐up.

Figure 3a–d shows the patients' joint pain VAS, tender joint count, swollen joint count, and the CRP value at baseline, post‐GMA, and at the 3‐month follow‐up. The patients' joint pain VAS score decreased from 75.1 ± 24.4 at baseline to 36.1 ± 36.7 post‐GMA; however, the difference was non‐significant (*p* = 0.075). Nevertheless, a noteworthy and statistically significant decrease was observed at the 3‐month follow‐up, at which the VAS score was 19.4 ± 21.7 (*p* = 0.029) (Figure 3a). The tender joint count decreased more than 50% from 7.6 ± 8.8 at baseline to 3.8 ± 3.4 post‐GMA and 3.5 ± 3.9 at the 3‐month follow‐up. However, these changes were statistically non‐significant (*p* = 0.258 and 0.063 respectively) (Figure 3b). Similarly, the swollen joint count decreased from 4.9 ± 8.0 at baseline to 3.9 ± 8.1 post‐GMA and 4.0 ± 8.8 at the 3‐month follow‐up. Nevertheless, these changes were also statistically non‐significant (*p* = 0.094 and 0.109 respectively) (Figure 3c). Additionally, the CRP level decreased significantly from 1.2 ± 2.1 mg/dL at baseline to 0.3 ± 0.3 mg/dL post‐GMA (*p* = 0.0002). However, at the 3‐month follow‐up, the CRP level increased in one patient due to a suspected iliopsoas abscess, resulting in a mean value of 1.6 ± 3.4 (Figure 3d).

**FIGURE 4:**
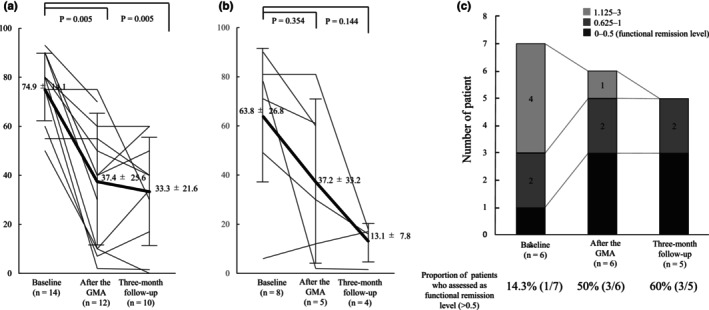
(a) Shifts in the visual analog scale (VAS) of the physician's global assessment (mm). (b) Shifts in the VAS of the patient's global assessment (mm) at each assessment time point. The solid lines represent individual patients, and the bold line represents the mean value for all patients. (c) Assessment of the Health Assessment Questionnaire at baseline after granulocyte and monocyte adsorptive apheresis (GMA) and at 3‐month follow‐up.

Furthermore, among the 10 patients assessed at the 3‐month follow‐up, one (10%) had significantly improved, and five (50%) had improved symptoms, for a total of six (60%) patients who were assessed as remarkably improved or improved (Figure 3e). Moreover, the VAS of the PGA decreased significantly from 74.9 ± 14.1 at baseline to 37.4 ± 25.6 post‐GMA (*p* = 0.005) and to 33.3 ± 21.6 at the 3‐month follow‐up (*p* = 0.005) (Figure 4a). Conversely, the VAS of the patients' global assessment decreased from 63.8 ± 26.8 at baseline to 37.2 ± 33.2 post‐GMA and to 13.1 ± 7.9 at the 3‐month follow‐up. However, these changes were statistically non‐significant (*p* = 0.354 and 0.144, respectively) (Figure 4b). The HAQ was administered to only seven patients at baseline, six patients post‐GMA, and five patients at the 3‐month follow‐up due to procedural issues. Of the patients who were able to be assessed using the HAQ, 50% (3/6) and 60% (3/5) achieved functional remission post‐GMA and at the 3‐month follow‐up, respectively (Figure 4c).

The efficacy of the treatment in improving skin and joint symptoms was evaluated based on the patients' smoking status and concurrent focal infections. The improvement rate of skin symptoms by smoking status was 0%, 28.6%, and 66.7% in the current smoking (0/2), cessation (2/7), and no smoking history group (2/3), respectively. The joint symptom rate was 0%, 85.7%, and 66.7% in the current smoking (0/2), cessation (6/7), and no smoking history group (2/3), respectively. No statistically significant difference was found in the improvement rate of the skin and joint symptoms among the three groups. Furthermore, when the cessation smoking group and no smoking history group were conflated into a no smoking group, the improvement rate was 25% (4/10) and 75% (8/10) for skin symptoms and joint symptoms, respectively. But no significant difference with the non‐smoking group was found (Table 2).

**TABLE 2:** Patient background and efficacy of GMA (*n* = 12).

	Skin manifestation			Joint symptom		
	Improved group (improvement rate^a^) (%)	Not improved group	*p*‐value	Improved group (improvement rate^a^) (%)	Not improved group	*p*‐value
Current smoking group (*n* = 2)	0 (0)	2	N.S.	0 (0)	2	N.S.
No smoking group (*n* = 10)	4 (40)	6		8 (80)	2	
Focal infection group (*n* = 5)	2 (40)	3	N.S.	4 (80)	1	N.S.
Non‐focal infection group (*n* = 7)	2 (28.6)	5		4 (57.1)	3	

*Note*: *p* ‐value by Fisher’s exact test analysis. Abbreviations: GMA, granulocyte and monocyte adsorptive apheres: N.S., not significant.

^a^The improvement rate was defined as the proportion of patients assessed as remarkably improved or improved. Improved group: Remarkable improved or Improved, Not improved group: Unchanged or deteriorated.

### 3.3 Safety

Among the 14 patients who underwent GMA once or more, two discontinued treatments due to adverse events. Hyperventilation was observed in one patient, possibly due to the stress of repeated punctures, but the patient eventually recovered. Another patient experienced joint pain, diarrhea, nausea, and dizziness, which were considered to be related to an underlying disease or a cold. However, this patient declined further treatment for personal reasons, and the outcome remains uncertain.

## 4 DISCUSSION

Kanekura et al. reported that GMA was effective for both skin and joint symptoms in PPP with PAO.^19^, ^21^, ^22^ Furthermore, a monocentric study by Sakanoue et al. reported that among 10 patients with PPP with joint symptoms, seven (70%) experienced improved or remarkably improved skin and/or joint symptoms.^20^ Similarly, a monocentric study by Kawakami et al. reported that of five PPP patients with skin manifestations, one (20%) experienced an improvement in skin manifestations after GMA, and of 10 PPP patients with joint symptoms, two (20%) had improved or remarkably improved joint symptoms (Table 3).^23^ In the present study, 33.3% (4/12) and 66.7% (8/12) of the patients experienced remarkable improvement or improvement in their skin and joint symptoms, respectively, post‐GMA. The results of the present, multicentric study were consistent with those of previous case reports and monocentric studies. Furthermore, Kawakami et al. reported that at a 3‐month follow‐up, 40% (2/5) and 50% (5/10) of patients with skin manifestations and joint symptoms, respectively, maintained a remarkably improved or improved condition.^23^

**TABLE 3:** Studies on GMA efficacy in PPP with PAO.

First author	Reporting year	No. of patients	Age, years	Sex	Efficacy for skin symptoms	Efficacy for joint symptoms	References
Kanekura	2003	1	57	F	Remarkably improved	Improved	^19^
Sakanoue	2013	10	35–62	F, 6; M, 4	Remarkably improved: 2 patients (20%)	Remarkably improved: 1 patient (10%)	^20^
					Improved: 5 patients (50%)	Improved: 7 patients (70%)	
Fujisawa	2014	1	61	M	Remarkably improved	Improved	^21^
Arimura	2018	1	47	M	Remarkably improved	Improved	^22^
Kawakami	2019	10	38–77	F, 9; M, 1	Post‐GMA, 1 of 5 patients (20%) had improved skin symptoms	Post‐GMA treatment, 2 patients (20%) had remarkably improved joint symptoms	^23^
					At 3 months, 2 of 5 patients (40%) had remarkably improved skin symptoms	At 3 months, 1 patient (10%) had remarkably improved, and 4 patients had improved, joint symptoms (40%)	

Abbreviations: F, female; GMA, granulocyte and monocyte adsorptive apheresis; M, male; PAO, pustulotic arthro‐osteitis; PPP, palmoplantar pustulosis.

In the present study, 70% (7/10) and 60% (6/10) of patients at the 3‐month follow‐up had remarkably improved or improved skin and joint symptoms, respectively. As in the study by Kawakami et al., some of the patients experienced the continued efficacy of GMA for a certain period after its completion or a gradual manifestation of GMA efficacy.

GMA is recognized for its ability not only to deplete activated granulocytes and monocytes, but also to modify their cell functions during their passage through the column. Patients with active GPP reportedly have an abundance of inflammatory monocytes (CD14 + CD16+ cells), which significantly decrease post‐GMA.^29^ Furthermore, the production of chemokines, including CCL3 (macrophage inflammatory protein [MIP]‐1α and CCL4 [MIP‐1β]), which are secreted by monocytes and promote the migration of neutrophils and monocytes, also decreases.^30^ GMA is thought to exert its therapeutic effects through various mechanisms, including changes in the functionality of passaging cells, suppression of inflammatory cytokine production, and the induction of anti‐inflammatory factors, regulatory T‐cells, and myeloid‐derived suppressor cells with immunoregulatory properties.^28^, ^29^, ^30^, ^31^, ^32^, ^33^, ^34^, ^35^, ^36^, ^37^ These mechanisms collectively ameliorate the pathophysiological condition. The mechanism underlying the continued efficacy of GMA is believed to be related to various immunomodulatory activities, including the inhibition of chemokines and inflammatory cytokines,^28^, ^29^, ^30^, ^31^, ^32^ and the induction of anti‐inflammatory factors^32^, ^35^, ^37^ and immunosuppressive cells.^32^, ^33^ This persistence of the therapeutic effects as well as the gradual manifestation of efficacy, may be attributable to these mechanisms. The present study did not include an in‐depth analysis of the characteristics differentiating patients with a sustained therapeutic effect from those with a gradual manifestation of efficacy. The present study investigated the impact of the previously uninvestigated factors of current smoking and the presence of concurrent focal infections on GMA efficacy. As Table 2 shows, unlike the findings reported for other pharmacologics,^8^, ^9^, ^10^ no significant difference in the efficacy of GMA for skin and joint symptoms was found regardless of smoking status and the presence of concurrent focal infections. It is worth noting that the relatively small sample size may have prevented an accurate determination of statistical significance. Nonetheless, the present study found that GMA might be effective regardless of smoking status or the presence of concurrent focal infections. At present, Japanese patients with psoriasis for whom conventional systemic treatments, including phototherapy, are ineffective are eligible for biologic and JAK inhibitor therapy under the National Health Insurance. Conversely, due to its high safety profile, GMA is used to treat GPP and PsA before the initiation of various biologics and JAK inhibitors or is administered to patients who are resistant to these treatments.^16^, ^17^, ^38^

In the present study, GMA was administered to patients with PPP with PAO in whom conventional systemic treatments, including NSAIDs and DMARDs, were ineffective or insufficient. However, since biologics had not been approved for use as a PPP treatment at the outset of this study and JAK inhibitors have yet to be approved, patients receiving these therapies were not included. Consequently, the efficacy and safety of GMA in patients with PPP with resistance or an inadequate response to biologics or JAK inhibitors remain unknown.

One of the strengths of this study is that it was prospective, observational, and conducted at three dermatology departments in Japan. Thus, the findings are likely to be more reliable than those in previous reports. However, the present study has several limitations. It was a single‐arm trial with no control group and a small sample size; thus, no definitive conclusions about the efficacy of GMA were able to be drawn. Imaging studies were not performed on any patients following the administration of the GMA. Accordingly, no comparisons were made between the pre‐ and post‐treatment data. Moreover, owing to the relatively small sample size, this study has low statistical power, and larger studies are necessary to confirm its findings. Additionally, factors were not analyzed for their ability to predict therapeutic efficacy, and no imaging study, immunological, or histological analysis was conducted to investigate the persistence of the treatment effect. These areas warrant further investigation.

Despite these limitations, the present study found that GMA may be an effective treatment for PPP with PAO, particularly in cases where conventional, systemic treatments are ineffective or poorly tolerated. This study also found no severe adverse effects associated with GMA use, indicating that it is a relatively safe treatment. While new medications for PPP may be developed, currently, the favorable safety profile of GMA makes it suitable for a variety of patients, including those undergoing screening before the initiation of biological therapy, pregnant or breastfeeding patients, children, the elderly, and those with a concurrent medical condition. Furthermore, GMA can be used concomitantly with conventional treatments, making it a safe therapeutic option for patients with PPP.

In conclusion, the present study found potential benefits of GMA using Adacolumn® in patients with PPP and PAO. Further research is required to corroborate these findings, confirm the efficacy of GMA in patients who are unresponsive to biological therapy, elucidate the mechanisms underlying its effects, and shed light on the long‐term impact of GMA on disease progression and quality of life.

## CONFLICT OF INTEREST STATEMENT

None declared.

Daiske Tsuruta is an Editorial Board member of Journal of Dermatology and a co‐author of this article. To minimize bias, he was excluded from all editorial decision‐making related to the acceptance of this article for publication.
